# Efficiency and Clinical Utility of AI-Assisted Radiotherapy Planning Using RatoGuide for Oropharyngeal Cancer: A Case Report

**DOI:** 10.7759/cureus.78388

**Published:** 2025-02-02

**Authors:** Yojiro Ishikawa, Kengo Ito, Satoshi Teramura, Takayuki Yamada

**Affiliations:** 1 Radiology, Tohoku Medical and Pharmaceutical University, Sendai, JPN

**Keywords:** adaptive radiotherapy, artificial intelligence (ai), deep learning artificial intelligence, intensity modulated radiation therapy (imrt), radiotherapy planning, ratoguide, volumetric‐modulated arc therapy (vmat)

## Abstract

This study evaluates the efficiency and dosimetric performance of RatoGuide, an artificial intelligence (AI)-assisted radiotherapy planning tool, by comparing AI-generated and manually created treatment plans for a 50-year-old male with right-sided oropharyngeal cancer (cT2N2bM0, cStage IVA) who underwent concurrent chemoradiotherapy. Treatment plans were created using volumetric-modulated arc therapy (VMAT) following the approach used by the Japanese Clinical Oncology Group (JCOG) protocol. RatoGuide generated two plans: one prioritizing the planning target volume (PTV) and the other focusing on organs at risk (OAR), while an experienced radiation oncologist manually developed a plan using a treatment planning system (TPS). Dosimetric comparisons focused on target coverage, OAR sparing, and dose homogeneity. Results showed that both AI-generated and TPS plans achieved comparable PTV coverage, with nearly identical values for Dmin, Dmean, and Dmax. The TPS plan exhibited slightly better dose homogeneity, whereas the AI-generated plan provided superior OAR sparing, particularly for the spinal cord and parotid glands, reducing the spinal cord’s intermediate-dose volume (V30) by approximately 40%. However, the AI plan yielded slightly higher mean doses to both submandibular glands, though still within clinically acceptable thresholds. Additionally, the AI planning workflow was completed in just 30 minutes, significantly reducing the time required for manual planning. RatoGuide demonstrated efficiency in generating high-quality treatment plans, achieving comparable PTV coverage, and improving OAR sparing in certain areas. However, minor refinements are needed to optimize dose homogeneity and further minimize submandibular gland exposure. These findings suggest that AI-assisted planning has the potential to enhance radiotherapy efficiency and consistency.

## Introduction

Radiotherapy treatment planning is a critical process aimed at maximizing therapeutic effects while minimizing radiation exposure to normal tissues [1]. Individualized approaches that account for patient-specific factors, such as tumor geometry and organ sensitivities, are essential to achieve precise dose distribution, enhancing efficacy while reducing side effects [2]. However, traditional treatment planning often relies heavily on the expertise of medical professionals, requiring extensive time and effort, with variability in plan quality posing a significant challenge [3,4]. In recent years, artificial intelligence (AI)-assisted tools have emerged as promising innovations for enhancing the efficiency and standardization of radiotherapy planning. Deep learning techniques, particularly those applied to intensity-modulated radiotherapy (IMRT) and volumetric-modulated arc therapy (VMAT), have shown potential in predicting dose distributions, reducing planning times, and standardizing quality [5,6]. These advancements hold promise for mitigating the limitations of traditional approaches, including prolonged planning processes and inter-planner variability [7].

Among AI-driven solutions, RatoGuide is a tool designed to streamline and standardize radiotherapy planning by leveraging clinical data and AI-based prediction models [8]. This software automates contouring and dose distribution predictions, significantly reducing planning times. Although currently approved only for research and educational purposes in Japan, RatoGuide has demonstrated its feasibility and potential in studies involving lung cancer, and head and neck cancers [8,9]. This study presents a simulation-based application of RatoGuide in planning radiotherapy for a patient with oropharyngeal cancer. By analyzing the AI-generated plan, this report explores its efficiency, dosimetric advantages, and potential for clinical utility while addressing its limitations and ethical considerations.

## Case presentation

**Figure 1:**
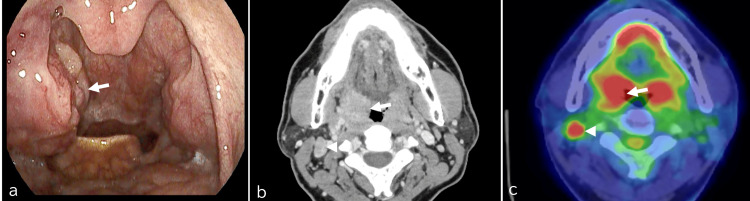
Endoscopic findings of the oropharynx and neck imaging in a case of oropharyngeal cancer. a.The laryngoscopic findings revealed enlargement of the right tonsil (arrow), b. Contrast-enhanced CT imaging demonstrated hypertrophy of the right tonsil (arrow) along with swelling of the cervical lymph node (arrowhead), c. PET-CT showed significant FDG uptake in the right tonsil (arrow) and cervical lymph node (arrowhead), indicating hypermetabolic activity consistent with malignancy.

The patient, a 50-year-old male, was diagnosed with right-sided oropharyngeal cancer classified as p16-positive cT2N2bM0 and staged as cStage IVA (Figure 1). He underwent concurrent chemoradiotherapy consisting of whole-neck irradiation at a total dose of 40 Gy delivered in 20 fractions, followed by a boost of 30 Gy in 15 fractions. Weekly cisplatin was administered throughout the radiotherapy course to enhance therapeutic efficacy.

The treatment targets were meticulously defined by a radiation oncologist with over 10 years of clinical experience to optimize coverage of malignant areas while minimizing radiation exposure to surrounding normal tissues. The gross tumor volume (GTV) included the primary tumor in the right tonsil and metastatic lymph nodes in the right level II-III regions. A clinical target volume (CTV) was established by adding a 0.5 cm margin to the GTV to account for potential microscopic disease. Prophylactic irradiation areas encompassed the bilateral level II-IVa, right level Ib, and right level Va-b lymph nodes to prevent further regional disease progression. To address potential setup uncertainties and organ motion, a planning target volume (PTV) was defined by adding an additional 0.5 cm margin to the CTV. OARs identified for dose sparing included the spinal cord, brainstem, parotid glands, submandibular glands, and mandible. The treatment plan was planned using VMAT with a dose prescription model of D50, where 50% of the prescribed dose was targeted to the PTV. The final dose calculation and optimization for all plans were performed using the RayStation treatment planning system (RaySearch Laboratories, Sweden).

The AI-assisted planning process utilized RatoGuide, an advanced software designed to automate contouring and optimize dose distribution. Digital imaging and communications in medicine (DICOM) data from the TPS were imported into RatoGuide, which applied a protocol aligned with the Japanese Clinical Oncology Group (JCOG) 1912 guidelines for head and neck cancer [10,11]. Using its pre-trained AI model, RatoGuide generated dose predictions and created optimization regions of interest (ROIs) for treatment planning. For the initial phase of 40 Gy whole-neck irradiation, RatoGuide produced two plans: a PTV-prioritized plan and an OAR-prioritized plan (Video 1).

**Video 1:** AI-assisted and human TPS plans for oropharyngeal cancer: whole-neck irradiation dose distributions. For the initial phase of 40 Gy whole-neck irradiation, the video compares two plans generated by RatoGuide: a PTV-prioritized plan and an OAR-prioritized plan. We recommend watching the video in 720p or higher for the best viewing experience.

**Figure 2:**
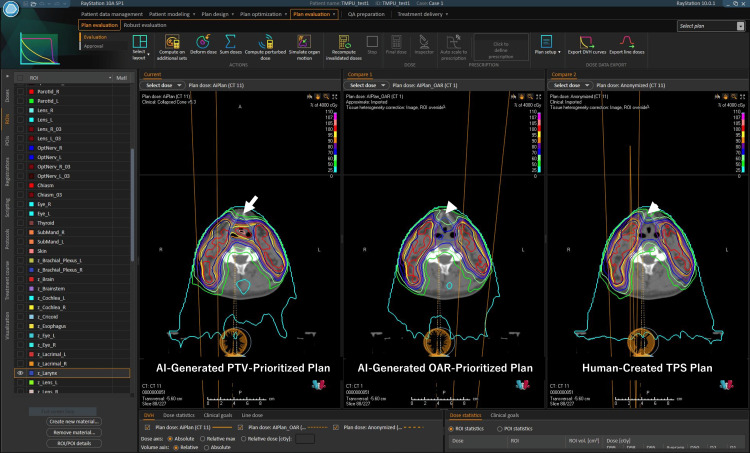
AI-assisted and human TPS plans for oropharyngeal cancer: whole-neck irradiation dose distributions. In the AI-generated PTV-prioritized plan, the dose to the larynx was elevated (arrow). In both the AI-generated OAR-Prioritized Plan and the human-created TPS Plan (arrowheads), there were no high-dose regions in the larynx, and the dose distribution appeared visually similar in these plans.

The PTV-prioritized plan achieved excellent dose coverage within the target volume but introduced hotspots in the larynx, which posed a potential risk of increased toxicity (Figure 2).

Consequently, the OAR-prioritized plan was selected for refinement, ensuring better sparing of critical structures. Based on this refined plan, the boost plan for the remaining 30 Gy was subsequently developed, maintaining adequate PTV coverage while prioritizing OAR protection. The AI-assisted planning workflow was completed in approximately 30 minutes, demonstrating its efficiency. The dosimetric comparison revealed that both the AI-generated and TPS plans achieved comparable target coverage within the PTV. Although dose uniformity was slightly better in the TPS plan, the AI plan demonstrated superior sparing of critical structures, such as the spinal cord and parotid glands. For instance, the spinal cord received a notably lower volume of intermediate doses in the AI plan, while the parotid glands experienced reduced mean doses, contributing to improved sparing of salivary gland function. However, the AI plan delivered slightly higher mean doses to both submandibular glands, which, while within clinically acceptable thresholds, may require further optimization to minimize potential long-term effects on salivary function (Video 2).

**Video 2:** AI-assisted and human TPS plans for oropharyngeal cancer: whole-neck and boost irradiation dose distributions The video compares an OAR-prioritized plan generated by RatoGuide with a treatment plan manually created by an experienced radiation oncologist. It includes dose distribution maps for the entire course of treatment, incorporating both the initial phase of 40 Gy whole-neck irradiation and the boost phase, reaching a total dose of 70 Gy. We recommend watching the video in 720p or higher for the best viewing experience.

These findings highlight the balance between efficiency and precision in AI-assisted radiotherapy planning, as detailed in Table 1.

**Table 1:** Comparison of dosimetric parameters between TPS and AI-generated plans. CI: conformity index; D1: dose received by the top 1% of the volume; D2: dose received by the top 2% of the volume; D50: dose covering 50% of the planning target volume; D98: dose received by the lowest 98% of the volume; Dmax: maximum dose; Dmean: mean dose; HI: homogeneity index; OAR: organ-at-risk; PTV, planning target volume; TPS: treatment planning system; V10: volume receiving 10 Gy; V20: volume receiving 20 Gy; V30: volume receiving 30 Gy; V40: volume receiving 40 Gy; V50: volume receiving 50 Gy.

Evaluation Item	TPS	AI	Unit/notes
Dmax (D0.01ml)	72.57	72.76	Maximum dose within 0.01ml
D1	71.73	71.51	The dose received by the top 1% of the volume identifies hotspots
D2	71.55	71.33	The dose received by the top 2% of the volume, near-maximum dose
Near-minimum dose (D98)	68.15	67.66	The dose received by the lowest 98% of the volume
Mean dose (Dmean)	70.18	70.01	Average dose across the entire PTV
Conformity index (CI)	0.95	0.96	Conformity of prescribed dose with PTV (ideal=1)
Homogeneity index (HI)	0.048	0.057	Uniformity of dose within the 50% dose region, HI=(D2-D98)/D50
Spinal cord maximum dose (Dmax)	35.53	34.92	≤45Gy (recommended limit)
V10 (spinal cord)	62.3	60	% (Volume of spinal cord receiving ≥10Gy)
V20 (spinal cord)	48	47.82	% (Volume of spinal cord receiving ≥20Gy)
V30 (spinal cord)	16.96	9.42	% (Volume of spinal cord receiving ≥30Gy)
Left parotid gland mean dose (Dmean)	23.87	21.51	≤26Gy
V40 (left parotid gland)	14.5	12.5	% (Volume of left parotid gland receiving ≥40Gy)
Right parotid gland mean dose (Dmean)	27.95	26.63	≤26Gy
V40 (right parotid gland)	22.5	20.6	% (Volume of right parotid gland receiving ≥40Gy)
Left submandibular dose (Dmean)	40.36	42.12	≤39 Gy (exceeds recommended limit)
Right submandibular dose (Dmean)	60.1	63.76	≤39 Gy (significantly exceeds recommended limit)
Mandible maximum dose (Dmax)	69.85	69.65	≤70Gy
Mandible mean dose (Dmean)	38.03	35.48	≤40–50 Gy
V50 (mandible)	25.61	25.23	% (Volume of mandible receiving ≥50Gy)

## Discussion

The findings of this case presentation are consistent with prior research demonstrating the effectiveness of deep learning-based tools in radiotherapy planning. For instance, Kadoya et al. highlighted the potential of deep learning to generate high-quality VMAT plans for prostate cancer, significantly improving clinical workflow efficiency while maintaining dosimetric precision [12]. Similarly, Saito et al. reported the feasibility of dose prediction in head and neck cancer cases using various contouring methods, underscoring the adaptability of AI-assisted planning for complex anatomical structures [13]. This case further explores the potential of RatoGuide, an AI-based tool for radiotherapy planning, emphasizing its ability to enhance planning efficiency and standardize quality across diverse clinical scenarios. Notably, RatoGuide is currently approved exclusively for research and educational purposes in Japan, limiting its application to non-clinical settings. Our report represents the first simulation-based study using RatoGuide for a real patient case, focusing on treatment planning for an oropharyngeal tumor. By simulating a clinical scenario, this study offers practical insights into how AI-assisted planning could be integrated into clinical practice in the future. Although these results are preliminary and do not establish clinical utility, they provide valuable data supporting future research and highlight the potential for broader applications once regulatory and technical hurdles are addressed.

In the comparison of dose distributions, both the AI-generated and human-created TPS plans provided comparable target coverage within the PTV, with similar values for minimum dose, mean dose, and maximum dose. These results indicate that both approaches achieved adequate dose coverage to the target area, meeting clinical objectives. Furthermore, when evaluating dose concentration, the CI for both plans was identical at a value of 1, demonstrating equivalent dose conformity to the PTV. This finding highlights that the AI-generated plan performs on par with the TPS plan in terms of dose concentration and coverage. The AI-generated plan also effectively reduced low-dose exposure to OARs, such as the parotid glands and mandible, while maintaining sufficient dose coverage within the PTV. Protecting salivary glands is particularly critical in head and neck cancer treatments, as sparing regions containing salivary stem cells have been shown to preserve saliva production and improve patients’ quality of life (QoL) after radiotherapy [14]. However, dose sparing for the submandibular glands was less stringent in the AI-generated plan. This limitation may stem from training data that prioritized other OARs over submandibular gland protection. Facilities that prioritize minimizing xerostomia-related side effects may need to incorporate manual adjustments to AI-generated plans to further optimize dose distributions for the submandibular glands [15,16]. These findings underscore the need to combine manual optimization with AI-driven planning, especially in cases where sparing specific OARs is crucial for patient outcomes. In terms of efficiency, RatoGuide completed the entire planning process in approximately 30 minutes. Compared to traditional methods, this represents a significant reduction in planning time, facilitating iterative optimization and rapid adjustments. The expedited planning process also underscores its potential application in adaptive radiotherapy, where swift re-planning is essential to accommodate anatomical changes during treatment [17].

Despite its promising performance, several challenges must be addressed to integrate AI-assisted planning systems like RatoGuide into clinical practice. One primary concern is the black-box nature of AI algorithms, where the rationale behind certain decisions, such as dose prioritization, may not be transparent to clinicians. This lack of explainability can hinder trust in AI-generated plans, particularly in cases involving unexpected complications or deviations from standard practices [18,19]. Efforts to develop explainable AI systems could mitigate these concerns by making AI outputs more interpretable and accessible [20]. Another limitation is the occurrence of hotspots outside the PTV in AI-generated plans, as observed in this case. These hotspots may increase the risk of adverse effects to adjacent normal tissues, necessitating further technical refinements to balance OAR protection with precise dose coverage. Cost considerations also arise, particularly if RatoGuide or similar AI tools are commercialized as high-cost proprietary software. Increased healthcare expenditures could limit accessibility, especially for smaller institutions with limited budgets. Affordability and equitable distribution of advanced AI-based planning tools will be critical to ensure widespread adoption [21]. The integration of AI in radiotherapy planning raises ethical questions about clinician involvement and patient safety. Over-reliance on AI could risk deskilling medical professionals and diminishing their critical role in the decision-making process [22]. In this case, while RatoGuide produced a high-quality plan, human oversight remained essential for adjustments and validation, emphasizing the importance of combining AI capabilities with clinical expertise. Additionally, as AI-based systems become more prevalent, regulatory frameworks must address the risks associated with software errors and system failures. Establishing robust validation protocols and guidelines will be essential to ensure the safe and effective implementation of AI in clinical practice.

## Conclusions

This case study highlights the potential of RatoGuide in improving radiotherapy planning efficiency and standardization, particularly for oropharyngeal cancer. The findings demonstrate its ability to reduce planning time, enhance dose distribution, and protect OARs. However, limitations such as the occurrence of hotspots, the black box nature of AI, and cost concerns must be addressed. While further research is necessary to validate its clinical utility, this report provides a foundational dataset for future studies and underscores the need for balanced integration of AI and clinician expertise in radiotherapy.
